# Impact of Chemotherapy Regimens on Normal Tissue Complication Probability Models of Acute Hematologic Toxicity in Rectal Cancer Patients Receiving Intensity Modulated Radiation Therapy With Concurrent Chemotherapy From a Prospective Phase III Clinical Trial

**DOI:** 10.3389/fonc.2019.00244

**Published:** 2019-04-09

**Authors:** Yikan Cheng, Yan Ma, Jian Zheng, Hua Deng, Xueqin Wang, Yewei Li, Xiaolin Pang, Haiyang Chen, Fang He, Lei Wang, Jianping Wang, Xiangbo Wan

**Affiliations:** ^1^Guangdong Provincial Key Laboratory of Colorectal and Pelvic Floor Diseases, Department of Radiation Oncology, Guangdong Institute of Gastroenterology, The Sixth Affiliated Hospital, Sun Yat-sen University, Guangzhou, China; ^2^Department of Radiation Oncology, Banner-University Medical Center Phoenix, Phoenix, AZ, United States; ^3^Department of Statistical Science, Southern China Center for Statistical Science, School of Mathematics, Sun Yat-sen University, Guangzhou, China; ^4^Zhongshan School of Medicine, Sun Yat-sen University, Guangzhou, China; ^5^Guangdong Provincial Key Laboratory of Colorectal and Pelvic Floor Diseases, Department of Colorectal Surgery, Guangdong Institute of Gastroenterology, The Sixth Affiliated Hospital, Sun Yat-sen University, Guangzhou, China

**Keywords:** rectal cancer, chemoradiotherapy, hematologic toxicity, FOWARC, normal tissue complication probability (NTCP)

## Abstract

**Purpose:** To determine whether there are differences in bone marrow tolerance to chemoradiotherapy (CRT) between two chemotherapy regimens according to FOWARC protocol and how chemotherapy regimens affect radiation dose parameters and normal tissue complication probability (NTCP) modelings that correlate with acute hematologic toxicity (HT) in rectal cancer patients treated with intensity modulated radiation therapy (IMRT) and concurrent chemotherapy.

**Materials and Methods:** One hundred and twenty-eight rectal cancer patients who received IMRT from a single institution were recruited from Chinese FOWARC multicenter, open-label, randomized phase III trial. We assessed HT in these patients who were separated into two groups: Oxaliplatin (L-OHP) + 5- fluorouracil (5FU) (FOLFOX, 70 of 128) and 5FU (58 of 128). The pelvic bone marrow (PBM) was divided into three subsites: lumbosacral spine (LSS), ilium (I), and lower pelvic (LP). The endpoint for HT was grade ≥3 (HT3+) and grade ≥2 (HT2+) leukopenia, neutropenia, anemia and thrombocytopenia. Logistic regression was used to analyze the association between HT2+/HT3+ and dosimetric parameters. Lyman-Kutcher-Burman (LKB) model was used to calculate NTCP.

**Results:** Sixty-eight patients experienced HT2+: 22 of 58 (37.9%) 5FU and 46 of 70 (65.7%) FOLFOX (*p* = 0.008), while twenty-six patients experienced HT3+: 4 of 58 (6.9%) 5FU and 22 of 70 (31.4%) FOLFOX (*p* = 0.016). PBM and LP dosimetric parameters were correlated with HT2+ in the 5FU group but not in the FOLFOX group. No PBM dosimetric parameters were correlated with HT3+ in both groups. For PBM, NTCP at HT3+ was 0.32 in FOLFOX group relative to 0.10 in 5FU subset (*p* < 0.05).

**Conclusion:** Patients receiving FOLFOX have lower BM tolerance to CRT than those receiving 5FU. Low-dose radiation to the PBM is predictive for HT2+ in patients who received 5FU. NTCP modeling in FOLFOX group predicts much higher risk of HT3+ than 5FU group.

## Introduction

Rectal cancer is a common malignancy in the world (1). Several randomized trials have demonstrated that preoperative radiotherapy (RT) combined fluorouracil (FU)-based chemotherapy improves locoregional control (LRC) in patients with locally advanced rectal cancer (2–5). Acute hematologic toxicity (HT) is a frequent complication of preoperative chemoradiotherapy (CRT) that can lead to prolongation of treatment time and suboptimal delivery of planned treatment (6, 7), which is detrimental for local tumor control.

Bone marrow (BM) tolerance to pelvic CRT in rectal cancer patients is poorly understood, although several studies indicated a correlation between low-dose radiation parameters to the pelvic BM (PBM) and acute HT in cervix (8–11) and anal cancer (12, 13) patients receiving intensity-modulated radiation therapy (IMRT). Moreover, results from Lyman-Kutcher-Burman (LKB) normal tissue complication probability (NTCP) modeling of PBM in anal and cervix cancer are consistent with a parallel-like structure of the PBM (10, 13). However, NTCP modeling of PBM for acute HT still remains unclear in rectal cancer.

On the other hand, acute HT risk depends on not only radiation dose but also chemotherapy regimens. Meta-analysis including 7 phase III trials indicates that the addition of oxaliplatin (L-OHP) to 5-fluorouracil (5FU) results in much higher rates of 3–4 grade acute toxicity compared with 5FU alone (14). Therefore, how different chemotherapy regimens affect radiation dose parameters and NTCP modeling that correlate with acute HT of PBM in rectal cancer need to be investigated.

To investigate whether differences in myelosuppressive activity of chemotherapy agents would alter the LKB parameters and the value of NTCP, we constructed NTCP models of acute HT in rectal cancer patients treated with IMRT based on two chemotherapy regimens (5FU vs. FOLFOX) received in the leading center from a phase III randomized clinical trial (FOWARC study: NCT01211210) (15). Furthermore, we aim to identify dosimetric parameters that correlate with HT in these two subgroups.

## Materials and Methods

### Patients

FOWARC is a multicenter, open-label, randomized, phase III study (15). Patients were randomly assigned (1:1:1) to receive neoadjuvant therapy with fluorouracil plus radiotherapy (5FU group), FOLFOX plus radiotherapy (FOLFOX group), or FOLFOX without radiotherapy followed by TME resection and post-operative adjuvant chemotherapy. Patients who completed radiotherapy in the leading center were included.

### Radiation Therapy

Patients in the leading center received 5-fields IMRT with an Elekta Synergy accelerator (with 80 MLCs) with 6 MV photon, delivered at 1.8–2.0 Gy per day per fraction from Monday to Friday for a total of 23–28 fractions and a total dose of 46.0–50.4 Gy. Pinnacle (version 9.0) was used for treatment planning. In Pinnacle, a collapsed cone convolution superposition model is used. The gross tumor volume (GTV) was defined as gross disease determined from MRI and CT or PET-CT. The clinical target volume (CTV) was defined as the GTV plus areas considered at significant risk of harboring microscopic disease, including the mesorectum (perirectal fascia), perirectal nodes, presacral region, and internal iliac lymph node region. External iliac nodes should be included when the primary tumor invades adjacent organs (cT4) or if the obturator nodes or external iliac nodes are involved. In addition, inguinal nodes should be included if the primary tumor directly invades the inguinal nodes or if the external anal sphincter is infiltrated. The planning target volume (PTV) was generated by adding an 8-mm margin around the CTV in all directions. The critical normal organs at risk (OARs) were the bladder, femoral heads, and small bowel. The dose of the OARs was set as low as possible and had to meet the following constraints: bladder, V50 ≤ 50%; femoral heads, V50 ≤ 5%; small bowel, V50 ≤ 5%.

### Chemotherapy

Patients in the 5FU group received preoperative treatment with five cycles of infusional fluorouracil (leucovorin 400 mg/m^2^ intravenously followed by fluorouracil 400 mg/m^2^ intravenously and fluorouracil 2.4 g/m^2^ by 48-h continuous intravenous infusion) with concurrent radiotherapy during cycles 2–4 and post-operative adjuvant chemotherapy with seven cycles of fluorouracil. Patients in the FOLFOX group received the same treatment as the 5FU group plus L-OHP 85 mg/m^2^ intravenously on day 1 of each chemotherapy cycle.

### PBM Delineation

**Figure 1:**
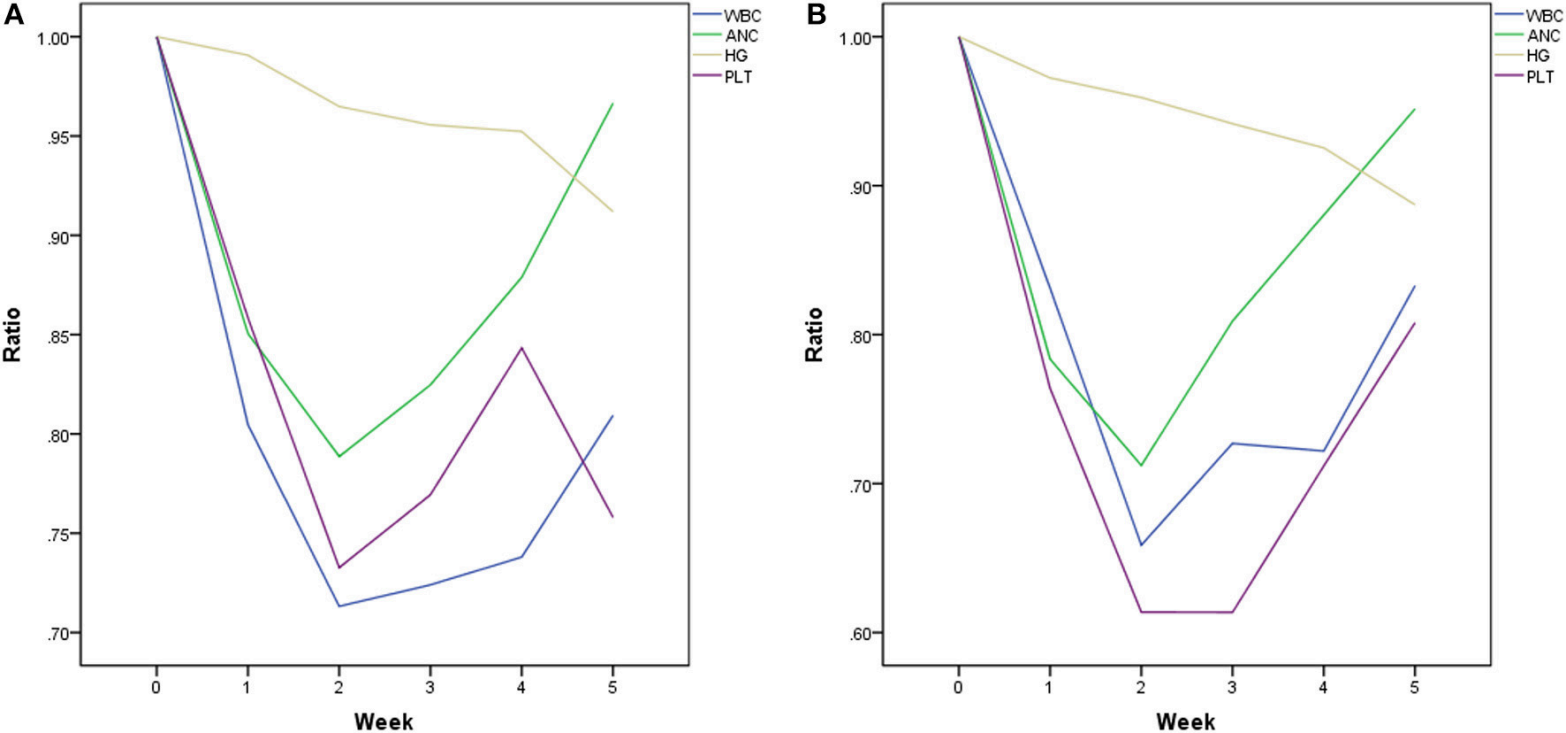
Trend of blood cell count ratio (mean): **(A)** 5FU group; **(B)** FOLFOX group.

An experienced radiation oncologist (Yikan Cheng) delineated PBM. The external contour of all bones within the pelvis was used as a surrogate for PBM, which was delineated on the planning CT. PBM was further divided into three subsites (9) (S Figure 1): (1) iliac BM (IBM), extending from the iliac crests to the superior border of the femoral head, (2) lower pelvis (LP), consisting of the pubes, ischia, acetabula, and proximal femora, extending from the superior border of the femoral heads to the inferior border of the ischial tuberosities, and (3) lumbosacral spine (LS), extending from the superior border of the L5 vertebral body to the coccyx but not extending below the superior border of the femoral head. Dose volume histograms (DVHs) were then generated for each contoured BM region. And the following parameters were recorded for PBM and each subsite: volume, mean dose, and the volume of each region receiving at least 5, 10, 15, 20, 30, and 40 Gy (V5, V10, V15, V20, V30, V40).

### Hematologic Toxicity

Complete blood counts were tested and collected weekly during concurrent chemoradiotherapy. HT was graded according to the Common Terminology Criteria for Adverse Events, version 3.0. The highest grade for white blood count, absolute neutrophil count, hemoglobin, and platelets was recorded for each patient. And the maximum severity score of all of these four HTs was recorded, with HT of grade ≥3 (HT3+) and grade ≥2 HT (HT2+) noted as endpoints.

### NTCP Modeling

The LKB model was used to represent NTCP and the value of NTCP was calculated using (Equations 1–4) (13, 16). E represents equivalent uniform dose (EUD) for PBM which calculated by the generalized Niemierko formula (16). The 3 parameters were used in the model: the volume parameter, n; the whole organ dose leading to a 50% complication risk, TD50; and a slope parameter, m. Maximum likelihood estimation was used to determine the optimum m, n, and TD50 with 95% confidence intervals (CI) for best fitting of calculated NTCP probabilities to the clinical data with constrained optimization of n (0<n≤1, unrestricted m and TD50).

(1) $$\begin{array}{l} NTCP\left(E\right)=e^{\left(AE-BE^{2}-C\right)} \end{array}$$

(2) $$\begin{array}{l} A=\left(\kappa +\frac{\kappa^{2}}{\text{m}}\right)\frac{1}{\text{m}TD50} \end{array}$$

(3) $$\begin{array}{l} B=\frac{\kappa^{2}}{{2\text{m}}^{2}{TD50}^{2}} \end{array}$$

(4) $$\begin{array}{l} C=ln2+\frac{\kappa }{m}+\frac{\kappa^{2}}{{2m}^{2}}=ln2-\frac{1}{2}+\frac{A^{2}}{4\text{B}} \end{array}$$

### Statistical Analysis

All analyses were conducted using STATA 12 statistical software (Stata Corp LP, College Station, Texas, USA). Distribution of baseline characteristics between two groups was evaluated using the χ2 test for categorical variables and *t*-test for continuous variables. Age, body mass index (BMI), and dosimetric parameters were coded as continuous variables. Univariate logistic regression was used to test the correlation between parameters and HT endpoints. Clinical parameters included sex, age, BMI, T classification, and N classification. Multivariate logistic regression models were then used to examine the effect of dosimetric parameters on HT. *p* < 0.05 was considered statistical significance.

## Results

### Patient Characteristics

**Figure 2:**
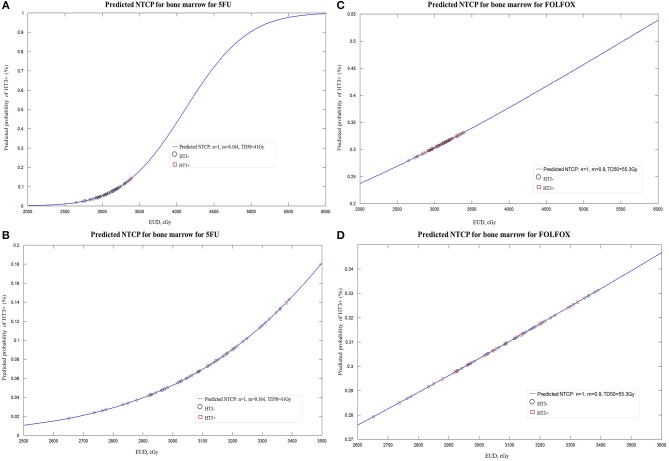
**(A)** Lyman-Kutcher-Burman normal tissue complication probability (NTCP) model for grade≥3 (HT3+) in patients treated with 5FU. Squares represent patients with HT3+. Open circles represent patients without HT3+. **(B)** Enlarged portion of **(A)**. **(C)** Lyman-Kutcher-Burman normal tissue complication probability (NTCP) model for grade≥3 (HT3+) in patients treated with FOLFOX. **(D)** Enlarged portion of **(C)**.

Of all 495 patients, 132 patients who received radiotherapy (5FUgroup and FOLFOX group) in the Sixth Affiliated Hospital, Sun Yat-sen University were included (S Figure 2). Four patients were excluded for damage of DICOM profiles. Eventually, 128 patients were included in our study, 58 patients in 5FU group and 70 patients in FOLFOX group. Patient baseline characteristics were well-balanced between two regimen groups (Table 1).

**Table 1:** Patient characteristics by chemotherapy regimen groups.

	**5FU (58)**	**FOLFOX (70)**	**All (128)**	***p*-value**
Sex (%)	38 (M, 65.5)	51 (M, 72.9)	89 (M, 69.5)	0.37
	20 (F, 34.5)	19 (F, 27.1)	39 (F, 30.5)	
Age (y), mean (*SD*)	56 (11)	52 (11)	54 (11)	0.008^*^
Comorbidity (%)	23 (39.7)	26 (37.1)	49 (38.3)	0.77
BMI(kg/m^2^), mean (*SD*)	23.2 (3.0)	23.1 (3.1)	23.1 (3.0)	0.90
T classification^#^ (%)				0.45
1–2	2 (1.7)	1 (1.4)	2 (1.6)	
3–4	57 (98.3)	69 (98.6)	126 (98.4)	
N classification^#^ (%)				0.79
0	11 (19.0)	12 (17.1)	23 (18.0)	
1–2	47 (81.0)	58 (82.9)	105 (82.0)	
Prescribed dose to primary tumor				0.40
Median, Gy	50	50	50	
Range, Gy	48–52	46–52	46–52	
Prescribed dose to pelvis				0.30
Median, Gy	46	46	46	
Range, Gy	45–46	45–46	45–46	

*5FU, 5- fluorouracil; FOLFOX, 5-fluorouracil+ oxaliplatin; SD, standard deviation; BMI, body mass index*.

* *Statistically significant*.

# *According to the 7th AJCC/UICC staging system*.

### Hematologic Toxicity

Baseline and nadir blood count values and HT rates were shown in Table 2 and the plots of longitudinal rates of blood counts were shown in Figure 1. Baseline blood count values were balanced quite well. However, nadir count values of WBC, ANC, Hemoglobin and Platelet between two chemotherapy regimen groups were significantly different, with *p* value of < 0.001, < 0.001, 0.025, and 0.001, respectively. Overall, the rate of HT2+ and HT3+ was significantly different between two groups: 37.9% 5FU vs. 65.7% FOLFOX (*p* = 0.008) and 6.9% 5FU vs. 31.4% FOLFOX (*p* = 0.016).

**Table 2:** Hematologic toxicity by chemotherapy regimens.

	**5FU**	**FOLFOX**	**All**	***p*-value**
**WBC**				
iWBC (k/uL), mean (SD)	6.5 (1.8)	5.9 (1.6)	6.1 (1.7)	0.053
n WBC (k/uL), mean (SD)	3.7 (1.2)	2.8 (0.9)	3.2 (1.1)	<0.001^*^
Grade 0, *n* (%)	23 (39.7)	7 (10.0)	30 (23.4)	
Grade 1, *n* (%)	16 (27.6)	21 (30.0)	37 (28.9)	
Grade 2, *n* (%)	16 (27.6)	28 (40.0)	44 (34.4)	
Grade 3, *n* (%)	2 (3.4)	13 (18.6)	15 (11.7)	
Grade 4, *n* (%)	1 (1.7)	1 (1.4)	2 (1.6)	
**ANC**				
iANC (k/uL), mean (SD)	3.9 (1.5)	3.5 (1.5)	3.7 (1.5)	0.176
nANC (k/uL), mean (SD)	2.4 (1.0)	1.6 (0.7)	1.9 (0.9)	<0.001^*^
Grade 0, *n* (%)	32 (55.2)	18 (25.7)	50 (39.1)	
Grade 1, *n* (%)	17 (29.3)	19 (27.1)	36 (28.1)	
Grade 2, *n* (%)	6 (10.3)	19 (27.1)	25 (19.5)	
Grade 3, *n* (%)	2 (3.4)	12 (17,1)	14 (10.9)	
Grade 4, *n* (%)	1 (1.7)	2 (2.9)	3 (2.3)	
**HEMOGLOBIN**				
iHemoglobin (g/L), mean (*SD*)	128 (16)	124 (18)	126 (17)	0.217
n Hemoglobin (g/L), mean (*SD*)	116 (16)	108 (20)	112 (19)	0.025^*^
Grade 0, *n* (%)	38 (65.5)	37 (52.9)	75 (58.6)	
Grade 1, *n* (%)	13 (22.4)	12 (17.1)	25 (19.5)	
Grade 2, *n* (%)	7 (12.1)	16 (22.9)	23 (18.0)	
Grade 3, *n* (%)	0 (0)	3 (4.3)	3 (2.3)	
Grade 4, *n* (%)	0 (0)	2 (2.9)	2 (1.6)	
**PLATELETS**				
i Platelets (k/uL), mean (*SD*)	223 (70)	232 (61)	228 (65)	0.404
n Platelets (k/uL), mean (*SD*)	145 (46)	119 (41)	131 (45)	0.001^*^
Grade 0, *n* (%)	50 (86.2)	45 (64.3)	95 (74.2)	
Grade 1, *n* (%)	5 (8.6)	20 (34.5)	25 (19.5)	
Grade 2, *n* (%)	2 (3.4)	5 (7.1)	7 (5.5)	
Grade 3, *n* (%)	0 (0)	0 (0)	0 (0)	
Grade 4, *n* (%)	1 (1.7)	0 (0)	1 (0.8)	
Any grade 2+	22 (37.9)	46 (65.7)	68 (53.1)	0.008^*^
Any grade 3+	4 (6.9)	22 (31.4)	26 (20.3)	0.016^*^

*5FU, 5-fluorouracil; FOLFOX, 5-fluorouracil+ oxaliplatin; WBC, white blood count; ANC, absolute neutrophil count; i, initial; SD, standard deviation; k/mL, 1,000 cells/mL; n, nadir; NS, not significant*.

* *Statistically significant*.

### BM Dosimetric Parameters

PBM and LP dosimetric parameters by chemotherapy group were summarized in Table 3 (LSS and ilium results in S Table 1). No significant difference was found in all BM dosimetric parameters between groups.

**Table 3:** Pelvic bone marrow and low pelvic descriptive statistics by treatment group.

	**5FU mean (*SD*)**	**FOLFOX mean (*SD*)**	**All mean (*SD*)**
**PELVIC BONE MARROW**			
Volume (mL)	1431 (227)	1416 (243)	1423 (235)
Mean (cGy)	3077 (176)	3133 (190)	3108 (186)
V5 (%)	98.5 (2.3)	99.2 (1.4)	98.9 (1.9)
V10 (%)	93.6 (4.7)	95.0 (4.7)	94.3 (4.7)
V15 (%)	90.4 (5.4)	91.9 (5.3)	91.2 (5.4)
V20 (%)	82.8 (5.7)	84.5 (5.8)	83.8 (5.8)
V30 (%)	49.7 (7.4)	50.8 (7.4)	50.3 (7.4)
V40 (%)	28.0 (6.3)	29.3 (6.4)	28.7 (6.4)
**LOW PELVIC**			
Volume (mL)	596 (101)	583 (118)	589 (111)
Mean (cGy)	2901 (176)	2899 (289)	2900 (243)
V5 (%)	98.6 (2.8)	99.2 (1.8)	98.9 (2.3)
V10 (%)	92.3 (8.3)	92.7 (10.1)	92.5 (9.3)
V15 (%)	88.4 (9.3)	89.0 (11.3)	88.8 (10.4)
V20 (%)	76.9 (9.6)	77.5 (11.1)	77.2 (10.4)
V30 (%)	35.9 (9.1)	35.8 (8.3)	35.9 (8.6)
V40 (%)	17.7 (6.1)	17.7 (5.7)	17.7 (5.9)

5FU, 5-fluorouracil; FOLFOX, 5-fluorouracil+ oxaliplatin; SD, standard deviation; Vx, volume of structure that receives greater than or equal to x Gy. ^*^Statistically significant. LSS and ilium results in S Table 1.

### Predictors of HT

Univariate logistic regression analyses were performed in 5FU and FOLFOX chemotherapy groups. The correlation between dosimetric/clinical parameters and HT grade was analyzed. However, no predictor was found to be correlated with HT3+ in both groups. The results of univariate logistic regression analysis for HT2+ were shown in Table 4. Age correlated with HT2+ in patients treated with 5FU (*p* = 0.043). In addition, LP dosimetric parameters (V10, V15) and PBM (V15) were highly associated with HT2+ in patients treated with 5FU (*p* = 0.026, *p* = 0.011, and *p* = 0.046, respectively). As for FOLFOX group, BMI and N classification were correlated with HT2+ (*p* = 0.026 and *p* = 0.027).

**Table 4:** Univariate logistic regression analysis for grade 2+ hematologic toxicity.

	**5FU** ***P*-value**	**FOLFOX** ***P*-value**
Age, y	0.95 (0.90–0.99), 0.043^*^	1.043 (0.996–1.093), 0.071
Sex (F vs. M)	2.455 (0.799–7.541), 0.117	1.106 (0.372–3.285), 0.856
BMI, per kg/m^2^	1.083 (0.902–1.300), 0.394	0.82 (0.69–0.98), 0.026^*^
N classification (N0 vs. N1-2)	1.261 (0.902–1.763), 0.176	0.71 (0.53–0.96), 0.027^*^
**PELVIC BONE MARROW**		
V15	0.90 (0.81–0.99), 0.046^*^	1.015 (0.928–1.110), 0.778
**LOW PELVIC**		
V10	0.92 (0.86–0.99),0.026^*^	1.007 (0.960–1.056), 0.776
V15	0.92 (0.86–0.98), 0.011^*^	1.000 (0.958–1.044), 0.998

*5FU, 5-fluorouracil; FOLFOX, 5-fluorouracil+ oxaliplatin; NS, not significant; Vx, volume of structure that receives greater than or equal to x Gy; NS, not significant*.

* *Statistically significant. Univariate logistic regression was used to test the correlation between parameters and grade 2+ hematologic toxicity. Parameters included sex, age, BMI, T classification, N classification, and dosimetric parameters. Table only showed the significant correlated parameters with HT2+ in both groups*.

We performed multivariate logistic regression models that included these dosimetric parameters and age for the 5FU group and BMI and N classification for the FOLFOX group. For patients in the 5FU group, PBM V15, LP V10, and V15 were significantly correlated with HT2+ when adjusting for age (Table 5). In the FOLFOX group, N classification maintained a significant correlation with HT2+ after adjusting for BMI (Table 5).

**Table 5:** Multivariate logistic regression analysis for grade 2+ hematologic toxicity.

	**5FU OR (95%CI)** ***P*-value**	**FOLFOX OR (95%CI)** ***P*-value**
BMI, per kg/m^2^		0.83 (0.69–0.99),0.045^*^
N classification		0.73 (0.54–0.99),0.046^*^
Age, y	0.94 (0.89–0.99),0.032^*^	
PBM-V15	0.89 (0.80–0.99),0.031^*^	
Age, y	0.94 (0.89–0.99), 0.039^*^	
LP-V10	0.92 (0.86–0.99),0.023^*^	
Age, y	0.94 (0.89–0.99), 0.035^*^	
LP-V15	0.91 (0.85–0.98), 0.008^*^	

*5FU, 5-fluorouracil; FOLFOX, 5-fluorouracil+ oxaliplatin; OR, odds ratio; Vx, volume of structure that receives greater than or equal to x Gy; PBM, pelvic bone marrow; LP, low pelvic*.

* *Statistically significant. Multivariate logistic regression models were used to examine the effect of dosimetric parameters on HT2+. Therefore, the correlation between significant dosimetric parameters and HT2+ after adjusting for age were performed in multivariate logistic regression*.

### LKB NTCP Modeling

One of 58 patients from 5FU group was excluded for NTCP modeling since the Excel of specific dosimetric data can't be correctly extracted from DICOM file. LKB modeling for PBM was performed in both regimen groups for HT3+ and in 5FU group for HT2+ but not in FOLFOX group (since the fitting didn't converge form up to 3). Constrained optimization of the LKB model for HT3+ and HT2+ yielded the value *n* = 1, confirming the expectation that over this restricted range characteristic of LKB tissue modeling, PBM acts like a parallel organ. The constrained maximum likelihood estimation optimization for PBM was performed in both groups. Constrained MLE optimization for HT3+ yielded parameters *n* = 1, *m* = 0.164, TD50 = 41.1 Gy for 5FU group and *n* = 1, *m* = 0.9, TD50 = 55.3 Gy for FOLFOX group (Figure 2). Increase in NTCP using parameter values in the FOLFOX group was statistically significant (*p* < 0.05). Mean NTCP at HT3+ was 0.32 in FOLFOX group relative to 0.10 in 5FU group. MLE optimization for HT2+ in 5FU group yielded parameters *n* = 1, *m* = 0.4852; TD50 = 36.8 Gy (S Figure 3).

## Discussion

While prior studies have shown both clinical and dosimetric predictors for HT in rectal cancer patients undergoing pelvic IMRT concurrent 5FU chemotherapy (7, 17), it is unclear which predictors correlate with HT in FOLFOX regimen and how different chemotherapy agents impact NTCP modeling of PBM in rectal cancer. To identify the myelosuppressive effect caused by different chemotherapy agents with IMRT, we investigate patients undergoing neoadjuvant IMRT with 5FU and FOLFOX for rectal cancer patients from a phase III trial (15). To our knowledge, this is the first study to use the LKB model to predict acute HT in rectal cancer patients receiving CRT from a prospective clinical trial.

Here we demonstrated that nadirs of all hematologic cell types in FOLFOX group were significantly lower than that in 5FU group given the similar baseline cell values between two groups. In addition, FOLFOX group had much higher ratio of grade HT2+ and HT3+ than that of 5FU, which demonstrated that L-OHP has been associated with more myelosuppressive properties. Even though there were no dosimetric predictors correlated with grade of HT2+ or HT3+, stricter dose constraints on PBM may be warranted given the myelosuppressive effect of L-OHP. Furthermore, we found a correlation between HT2+ and BMI in FOLFOX group, with a protective effect of higher BMI, which was consistent with a study by Jose et al. (18). This study indicated that higher BMI was associated with lower rates of HT in patients receiving CRT for pelvic cancers. Similarly, lower rates of HT among esophageal carcinoma patients correlated with high BMI (19). In addition, such trend was also found in patients receiving chemotherapy for colon (20) and breast (21) cancers. Therefore, BMI may be an important marker to consider for patients receiving L-OHP when evaluating their risk for developing HT. Another predictor for grade HT2+ in FOLFOX group was N classification, which may be explained by higher dose for malignant lymph node or larger target volume in target delineation.

Acute severe hematologic toxicity (HT3+) associated with pelvic RT and continuous infusion 5FU for rectal cancer has been reported up to 8% (18), which was similar to our result. As for grade 2+ leukopenia, the rate in 5FU group was 32.7%, which was much higher compared with Yang's study (7). This distinction may result from the majority of patients from Yang's study received 3D-conformal radiotherapy. Since IMRT treatment has been shown to significantly increase PBM irradiation according to Robinson et al. (22), our patients received IMRT were found to have higher rates of HT2+. Mell et al. (12) found that IMRT can increase low-dose radiation to PBM, which was associated with acute HT during chemoradiotherapy. Similarly, our analysis showed that PBM dosimetric predictors for HT2+ in 5FU group were PBM V15, LP V15, and V10. However, the study from Memorial Sloan Kettering Cancer Center showed coxal BM V45 and sacral BM V45 were associated with a lower WBC and ANC nadir (7). This, somewhat paradoxical finding, may be explained by different radiation techniques mainly used in these two studies.

Even though it is mathematically valid to allow *n* > 1 in the LKB model, clinically meaningful interpretation is hard, especially when the entire organ is not homogeneously irradiated, as is the case with PBM in our study (13). Therefore, with the common used approach of constraining n, the values were *n* = 1 for both 5FU and FOLFOX groups, implying that the PBM is a parallel structure. In our exploratory calculations with the LKB model for HT3+, we found that the NTCP modeling was predictive of a tripling of risk associated with FOLFOX, supporting the more myelosuppressive properties of L-OHP and suggesting that stricter dose constraints on PBM with FOLFOX may be warranted.

The study from Stanford University (18) suggested that PBM sparing may be less important for those patients receiving IMRT with concurrent 5FU because of its low rates of HT3+. However, Newman et al. (23) found that neoadjuvant 5FU based chemoradiotherapy for rectal cancer had long term bone marrow suppression during post-operative chemotherapy, which demonstrated that sparing of the PBM during preoperative chemoradiotherapy can aid in reducing significant hematologic adverse events and tolerance of post-operative chemotherapy. Similarly, this study supports the notion that sparing of the PBM should be applied in rectal cancer patients receiving IMRT, even though it is less important for those patients receiving radiation with 5FU alone because of the low rates of HT but important for patients receiving radiation and FOLFOX, who have much higher rates of HT3+. Dose constraints may be further tailored on the basis of specific chemotherapy regimen. Therefore, the present study supports the notion that sparing of the PBM can be tailored in a more patient-specific manner. Moreover, efforts to spare PBM are particularly important for patients with low BMI because they are at increased risk of developing HT.

To our knowledge, this is the first study to demonstrate that low dose to PBM in IMRT may be an important dosimetric parameter with regard to HT and build LKB NTCP models to predict acute HT from radiation to PBM in rectal cancer patients with two common chemotherapy regimens in a prospective clinical trial. However, our analysis has its limitation. Given that this was a single institution review of a relatively small cohort, our results can only be viewed as hypothesis generating, which will need to be validated in a larger collected group of data. Furthermore, we contoured the entire bone as opposed to the actual PBM, which had its own flaws: inter- and intra-subject variability. Another limitation was that we do not have any information on the function distribution of active bone marrow which can be defined by SPECT (24). The last limitation of our study was that the small number of patients in each group limits the refinement of our models further based on other factors, such as age and BMI. For instance, log-linear mixed effects models are efficient especially when covariates are time varying (25), which seems to fit our data structure. Therefore, further study using other modeling to validate our result is warranted.

## Conclusions

In conclusion, the incidence of HT2+ and HT3+ depends on type of chemotherapy regimen received in rectal cancer patients. Patients receiving IMRT with 5FU have low rates of HT3+ and HT2+. Patients treated with FOLFOX have lower BM tolerance to CRT than those treated with 5FU. Low-dose radiation to the PBM is predictive for HT2+ in patients who received 5FU but not FOLFOX. NTCP modeling in FOLFOX group predicts much higher risk of HT3+ than 5FU group. Chemotherapy-adapted dose constraints for the PBM should be developed by future prospective protocols.

## Ethics Statement

This study was approved by the central ethics committee of the Sixth Affiliated Hospital, Sun Yat-sen University (Guangzhou, China) and was conducted in accordance with the Declaration of Helsinki. Written informed consent was obtained from all enrolled patients.

## Author Contributions

YC, JW, and XBW designed this work of review. YC, YM, JZ, and HD coordinated subject inclusion, data handling, and data storage. HD, YL, and XQW analyzed statistics. XP, HC, and FH were involved in literature research. YC drafted the manuscript. LW, JW, and XBW revised the manuscript. All authors approved the paper for publication.

### Conflict of Interest Statement

The authors declare that the research was conducted in the absence of any commercial or financial relationships that could be construed as a potential conflict of interest.

## References

[1] Torre LA Bray F Siegel RL Ferlay J Lortet-Tieulent J Jemal A. Global cancer statistics, 2012. CA Cancer J Clin. (2015) 65:87–108. 10.3322/caac.21262 25651787

[2] Randomised trial of surgery alone versus radiotherapy followed by surgery for potentially operable locally advanced rectal cancer Medical Research Council Rectal Cancer Working Party. Lancet. (1996) 348:1605–10. 10.1016/S0140-6736(96)05348-2 8961989

[3] Swedish Rectal Cancer Trial Cedermark B Dahlberg M Glimelius B Påhlman L Rutqvist LE Improved survival with preoperative radiotherapy in resectable rectal cancer. Swedish Rectal Cancer Trial. N Engl J Med. (1997) 336:980–7. 10.1056/NEJM199704033361402 9091798

[4] Sauer R Becker H Hohenberger W Rödel C Wittekind C Fietkau R. Preoperative versus postoperative chemoradiotherapy for rectal cancer. N Engl J Med. (2004) 351:1731–40. 10.1056/NEJMoa040694 15496622

[5] Sauer R Liersch T Merkel S Fietkau R Hohenberger W Hess C. Preoperative versus postoperative chemoradiotherapy for locally advanced rectal cancer: results of the German CAO/ARO/AIO-94 randomized phase III trial after a median follow-up of 11 years. J Clin Oncol. (2012) 30:1926–33. 10.1200/JCO.2011.40.1836 22529255

[6] Fernández-Martos C Pericay C Aparicio J Salud A Safont M Massuti B. Phase II, randomized study of concomitant chemoradiotherapy followed by surgery and adjuvant capecitabine plus oxaliplatin (CAPOX) compared with induction CAPOX. followed by concomitant chemoradiotherapy and surgery in magnetic resonance imaging-defined, locally advanced rectal cancer: Grupo cancer de recto 3 study. J Clin Oncol. (2010) 28:859–65. 10.1200/JCO.2009.25.8541 20065174

[7] Yang TJ Oh JH Apte A Son CH Deasy JO Goodman KA. Clinical and dosimetric predictors of acute hematologic toxicity in rectal cancer patients undergoing chemoradiotherapy. Radiother Oncol. (2014) 113:29–34. 10.1016/j.radonc.2014.09.002 25304718 PMC4822505

[8] Albuquerque K Giangreco D Morrison C Siddiqui M Sinacore J Potkul R. Radiation-related predictors of hematologic toxicity after concurrent chemoradiation for cervical cancer and implications for bone marrow-sparing pelvic IMRT. Int J Radiat Oncol Biol Phys. (2011) 79:1043–7. 10.1016/j.ijrobp.2009.12.025 20471182

[9] Mell LK Kochanski JD Roeske JC Haslam JJ Mehta N Yamada SD. Dosimetric predictors of acute hematologic toxicity in cervical cancer patients treated with concurrent cisplatin and intensity-modulated pelvic radiotherapy. Int J Radiat Oncol Biol Phys. (2006) 66:1356–65. 10.1016/j.ijrobp.2006.03.018 16757127

[10] Rose BS Aydogan B Liang Y Yeginer M Hasselle MD Dandekar V. Normal tissue complication probability modeling of acute hematologic toxicity in cervical cancer patients treated with chemoradiotherapy. Int J Radiat Oncol Biol Phys. (2011) 79:800–7. 10.1016/j.ijrobp.2009.11.010 20400238 PMC2907446

[11] Liang Y Messer K Rose BS Lewis JH Jiang SB Yashar CM. Impact of bone marrow radiation dose on acute hematologic toxicity in cervical cancer: principal component analysis on high dimensional data. Int J Radiat Oncol Biol Phys. (2010) 78:912–9. 10.1016/j.ijrobp.2009.11.062 20472344 PMC2923677

[12] Mell LK Schomas DA Salama JK Devisetty K Aydogan B Miller RC. Association between bone marrow dosimetric parameters and acute hematologic toxicity in anal cancer patients treated with concurrent chemotherapy and intensity-modulated radiotherapy. Int J Radiat Oncol Biol Phys. (2008) 70:1431–7. 10.1016/j.ijrobp.2007.08.074 17996390

[13] Bazan JG Luxton G Mok EC Koong AC Chang DT. Normal tissue complication probability modeling of acute hematologic toxicity in patients treated with intensity-modulated radiation therapy for squamous cell carcinoma of the anal canal. Int J Radiat Oncol Biol Phys. (2012) 84:700–6. 10.1016/j.ijrobp.2011.12.072 22414279

[14] Yang YJ Cao L Li ZW Zhao L Wu HF Yue D. Fluorouracil-based neoadjuvant chemoradiotherapy with or without oxaliplatin for treatment of locally advanced rectal cancer: an updated systematic review and meta-analysis. Oncotarget. (2016) 7:45513–24. 10.18632/oncotarget.9995 27322422 PMC5216738

[15] Deng Y Chi P Lan P Wang L Chen W Cui L. Modified FOLFOX6 with or without radiation versus fluorouracil and leucovorin with radiation in neoadjuvant treatment of locally advanced rectal cancer: initial results of the chinese FOWARC multicenter, open-label, randomized three-arm phase III trial. J Clin Oncol. (2016) 34:3300–7. 10.1200/JCO.2016.66.6198 27480145

[16] Luxton G Keall PJ King CR. A new formula for normal tissue complication probability (NTCP) as a function of. equivalent uniform dose (EUD). Phys Med Biol. (2008) 53:23–36. 10.1088/0031-9155/53/1/002 18182685

[17] Wan J Liu K Li K Li G Zhang Z. Can dosimetric parameters predict acute hematologic toxicity in rectal cancer patients treated with intensity-modulated pelvic radiotherapy? Radiat Oncol. (2015) 10:162. 10.1186/s13014-015-0454-0 26238572 PMC4554292

[18] Bazan JG Luxton G Kozak MM Anderson EM Hancock SL Kapp DS. Impact of chemotherapy on normal tissue complication probability models of acute hematologic toxicity in patients receiving pelvic intensity modulated radiation therapy. Int J Radiat Oncol Biol Phys. (2013) 87:983–91. 10.1016/j.ijrobp.2013.09.017 24161422

[19] Wang J Myles B Wei C Chang JY Hofstetter WL Ajani JA. Obesity and outcomes in patients treated with chemoradiotherapy for esophageal carcinoma. Dis Esophagus. (2014) 27:168–75. 10.1111/dote.12074 23621168 PMC3740061

[20] Meyerhardt JA Catalano PJ Haller DG Mayer RJ Benson AB Macdonald JS. Influence of body mass index on outcomes and treatment-related toxicity in patients with colon carcinoma. Cancer Am Cancer Soc. (2003) 98:484–95. 10.1002/cncr.11544 12879464

[21] Griggs JJ Sorbero ME Lyman GH. Undertreatment of obese women receiving breast cancer chemotherapy. Arch Intern Med. (2005) 165:1267–73. 10.1001/archinte.165.11.1267 15956006

[22] Robinson M Sabbagh A Muirhead R Durrant L Van den Heuvel F Hawkins M. Modeling early haematologic adverse events in conformal and intensity-modulated pelvic radiotherapy in anal cancer. Radiother Oncol. (2015) 117:246–51. 10.1016/j.radonc.2015.09.009 26409831 PMC4678285

[23] Newman NB Sidhu MK Baby R Moss RA Nissenblatt MJ Chen T. Long-term bone marrow suppression during postoperative chemotherapy in rectal cancer patients after preoperative chemoradiation therapy. Int J Radiat Oncol Biol Phys. (2016) 94:1052–60. 10.1016/j.ijrobp.2015.12.374 27026312

[24] Roeske JC Lujan A Reba RC Penney BC Diane Yamada S Mundt AJ. Incorporation of SPECT bone marrow imaging into intensity modulated whole-pelvic radiation therapy treatment planning for gynecologic malignancies. Radiother Oncol. (2005) 77:11–17. 10.1016/j.radonc.2005.06.017 16024116

[25] Zhu X Qu A. Individualizing drug dosage with longitudinal data. Stat Med. (2016) 35:4474–88. 10.1002/sim.7016 27311930

