# Supplementary material for: Impact of Chemotherapy Regimens on Normal Tissue Complication Probability Models of Acute Hematologic Toxicity in Rectal Cancer Patients Receiving Intensity Modulated Radiation Therapy With Concurrent Chemotherapy From a Prospective Phase III Clinical Trial
Source: Front Oncol. 2019 Apr 9;9:244. doi: 10.3389/fonc.2019.00244 (PMC6465593; doi:10.3389/fonc.2019.00244)
Supplement: Supplementary file 1 [file Data_Sheet_1.docx]

S Table 1 LSS and ilium descriptive statistics by treatment group

|  | **5FU**  **Mean (SD)** | **FOLFOX**  **Mean (SD)** | **All**  **Mean (SD)** |
| --- | --- | --- | --- |
| LSS |  |  |  |
| Volume (mL) | 369 (65) | 363 (64) | 365 (64) |
| Mean (cGy) | 3817(334) | 3893 (481) | 3858 (421) |
| V5 (%) | 98.4 (3.8) | 99.5 (1.9) | 99.0 (2.9) |
| V10 (%) | 97.0 (5.1) | 98.6 (2.9) | 97.8 (4.1) |
| V15 (%) | 95.1 (5.8) | 97.3 (3.7) | 96.3 (4.9) |
| V20 (%) | 92.6 (6.6) | 95.1 (4.6) | 94.0 (5.7) |
| V30 (%) | 79.3 (9.8) | 82.2 (8.1) | 80.9 (9.0) |
| V40 (%) | 57.6 (10.5) | 60.3 (8.5) | 59.0 (9.5) |
| Illium |  |  |  |
| Volume (mL) | 466 (79) | 470 (78) | 468 (78) |
| Mean (cGy) | 2751 (231) | 2756 (285) | 2753 (261) |
| V5 (%) | 98.9 (2.1) | 98.9 (3.1) | 98.9 (2.6) |
| V10 (%) | 92.9 (5.0) | 94.3 (4.4) | 93.7 (4.7) |
| V15 (%) | 89.1 (5.8) | 90.6 (5.3) | 89.9 (5.6) |
| V20 (%) | 82.7 (6.6) | 84.2 (6.3) | 83.5 (6.5) |
| V30 (%) | 43.6 (8.2) | 43.6 (8.4) | 43.6 (8.3) |
| V40 (%) | 17.9 (6.3) | 18.3 (6.0) | 18.2 (6.2) |

Abbreviations: 5FU= 5-fluorouracil; FOLFOX= 5-fluorouracil+ oxaliplatin; LSS=lumbosacral spine subsite; Vx= volume of structure that receives greater than or equal to x Gy.

*statistically significant

S Figure 1. (A) Pelvic bone marrow subsites and tumor and (B) dose distribution of intensity modulated radiotherapy for rectal cancer in representative levels.

S Figure 2. Flow diagram of the study.

S Figure 3. (A) Lyman-Kutcher-Burman normal tissue complication probability (NTCP) model for HT2+ in patients treated with 5FU. Squares represent patients with HT2+. Open circles represent patients without HT2+. (B) Enlarged portion of (A).
