# Supplementary figures and images for: Impact of Chemotherapy Regimens on Normal Tissue Complication Probability Models of Acute Hematologic Toxicity in Rectal Cancer Patients Receiving Intensity Modulated Radiation Therapy With Concurrent Chemotherapy From a Prospective Phase III Clinical Trial
Source: Front Oncol. 2019 Apr 9;9:244. doi: 10.3389/fonc.2019.00244 (PMC6465593; doi:10.3389/fonc.2019.00244)

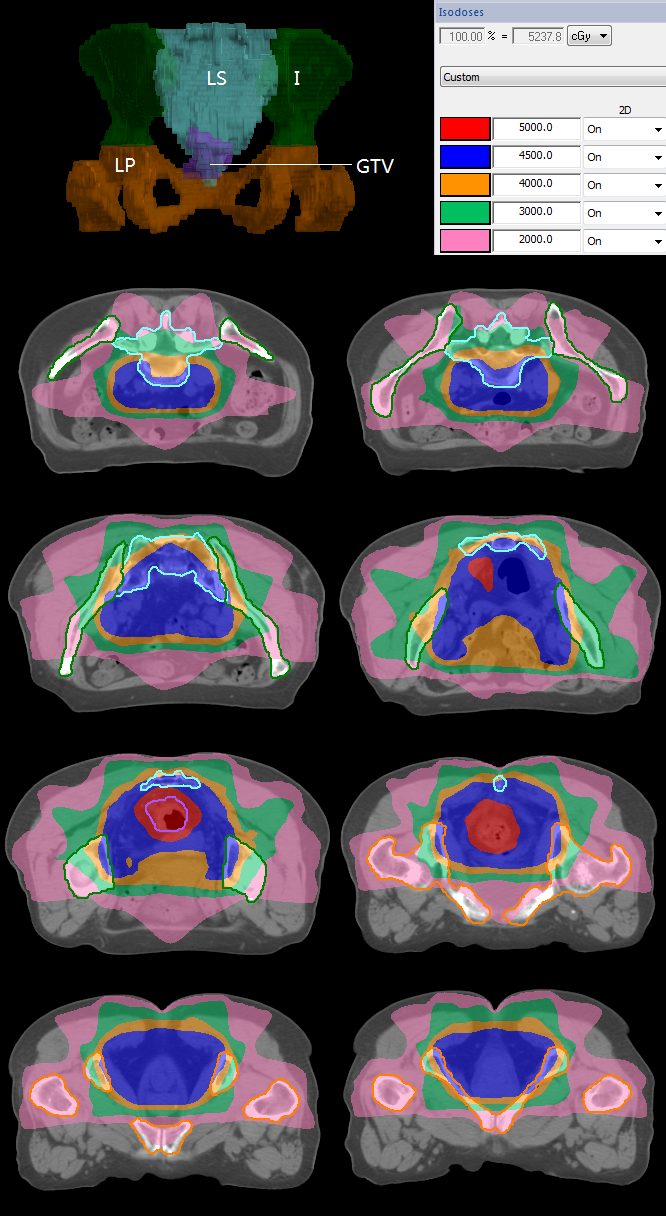

Supplement: Supplementary file 2 [file Image_1.tif]

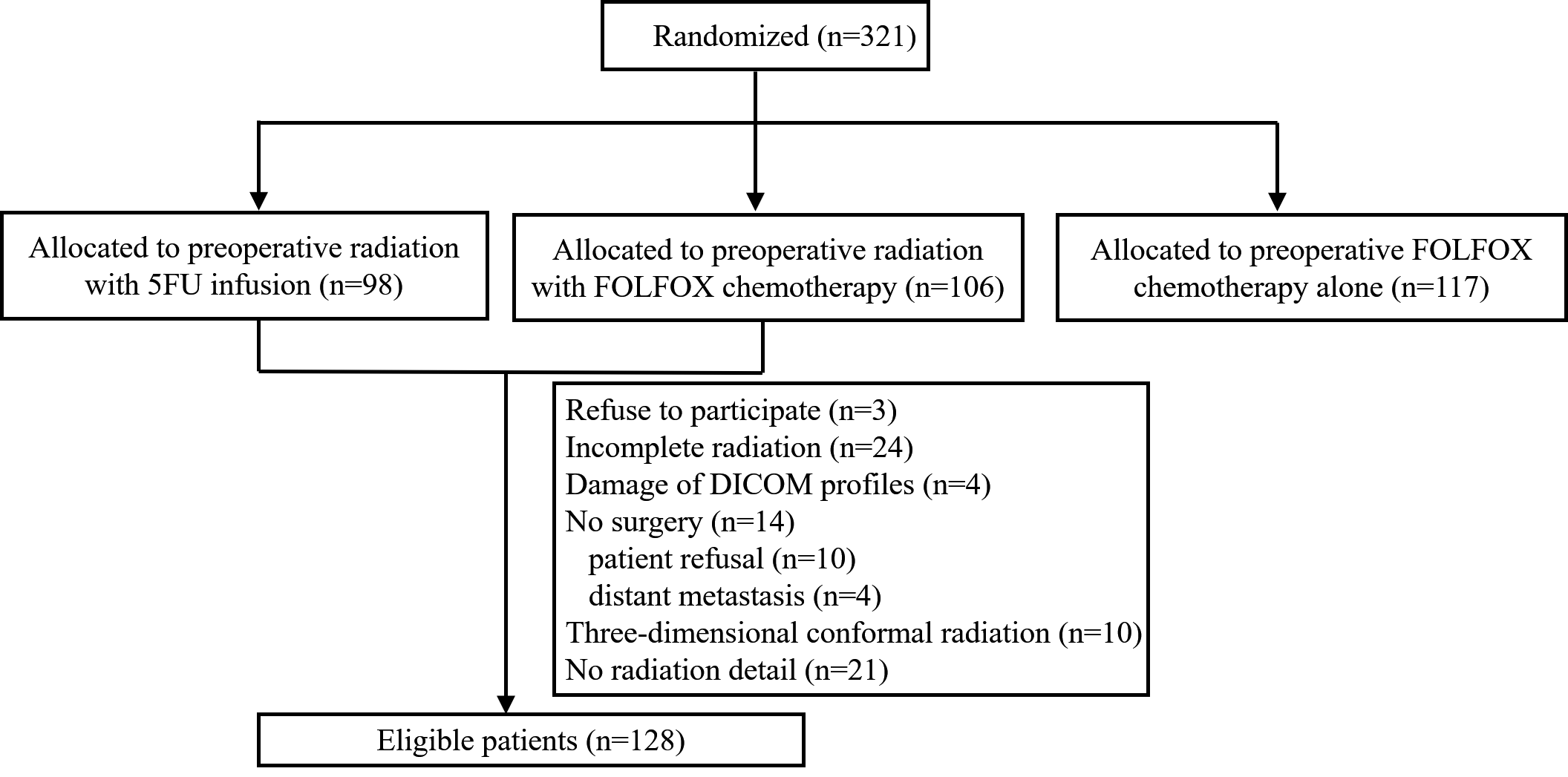

Supplement: Supplementary file 3 [file Image_2.tif]

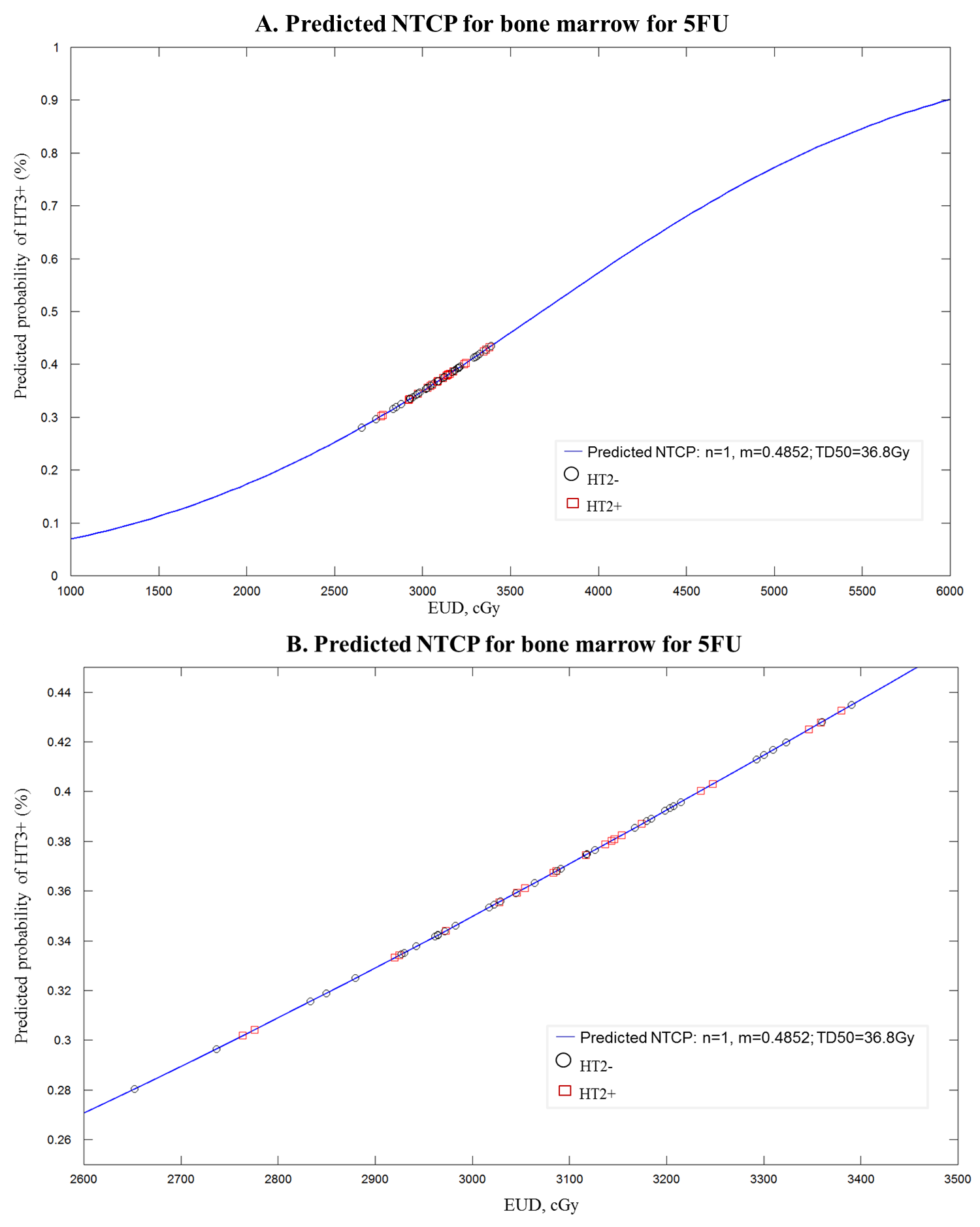

Supplement: Supplementary file 4 [file Image_3.tif]
